# Finding influential nodes in complex networks based on Kullback–Leibler model within the neighborhood

**DOI:** 10.1038/s41598-024-64122-4

**Published:** 2024-06-10

**Authors:** Guan Wang, Zejun Sun, Tianqin Wang, Yuanzhe Li, Haifeng Hu

**Affiliations:** 1 School of Information Engineering, Pingdingshan University, Pingdingshan, 467000 China; 2 Mechanical Department, Puyang Technician College, Puyang, 457000 China; 3 Baofeng County People’s Government, Pingdingshan, 467000 China

**Keywords:** Complex networks, Information dissemination, Influential nodes, Kullback–Leibler divergence, Neighborhood, Computational science, Computer science

## Abstract

As a research hot topic in the field of network security, the implementation of machine learning, such as federated learning, involves information interactions among a large number of distributed network devices. If we regard these distributed network devices and connection relationships as a complex network, we can identify the influential nodes to find the crucial points for optimizing the imbalance of the reliability of devices in federated learning system. This paper will analyze the advantages and disadvantages of existing algorithms for identifying influential nodes in complex networks, and propose a method from the perspective of information dissemination for finding influential nodes based on Kullback–Leibler divergence model within the neighborhood (KLN). Firstly, the KLN algorithm removes a node to simulate the scenario of node failure in the information dissemination process. Secondly, KLN evaluates the loss of information entropy within the neighborhood after node removal by establishing the KL divergence model. Finally, it assesses the damage influence of the removed node by integrating the network attributes and KL divergence model, thus achieving the evaluation of node importance. To validate the performance of KLN, this paper conducts an analysis and comparison of its results with those of 11 other algorithms on 10 networks, using SIR model as a reference. Additionally, a case study was undertaken on a real epidemic propagation network, leading to the proposal of management and control strategies for daily protection based on the influential nodes. The experimental results indicate that KLN effectively evaluates the importance of the removed node using KL model within the neighborhood, and demonstrate better accuracy and applicability across networks of different scales.

## Introduction

Various real-life systems can be regarded as different complex networks^1^, including marketing networks^2^, information dissemination networks^3^, urban transportation networks^4^, protein networks^5^, epidemic propagation networks^6^, electric power networks^7^ and so on. The algorithms for finding influential nodes are helpful to study and analyze the structures of real networks^8^. For instance, finding the influential communication objectives in marketing networks can accelerate the speed of marketing communication to obtain favorable income. Finding the influential communicators in information dissemination networks can effectively control the rumors and maintain a satisfactory environment for information dissemination. Finding the influential pivot points in urban transportation networks and electric power networks allows an effective evaluation of network robustness and invulnerability to avoid cascade accidents caused by the damage of some influential nodes. Finding the vital spreaders in epidemic propagation networks can effectively slow the spread and development of the epidemic, mitigate the dissemination speed of the virus, and reduce the scope of infection. As a hot research topic recently, federated learning is an emerging foundational technology in artificial intelligence, enabling multiple participants to collaboratively train models within a machine learning framework. This distributed learning mode results in complex connectivity and node distribution within the network^9^. In such an complex environment, we can regard this distributed network system as a complex network, thus identifying important nodes can also help us locate the nodes that significantly influence the entire system, and enhance the stability and resilience for federated learning. These show that the technology of finding influential nodes has been extensively used in various real networks in recently. Therefore, the algorithms for finding influential nodes are crucial for the study of real networks.

Scholars have been particularly interested in the design of accurate, fast, and efficient algorithms to identify influential nodes, with many algorithms having recently emerged. Classical algorithms include DC^10^, BC^11^, CC^12^, EC^13^, K-shell^14^, etc. However, these algorithms have various advantages and disadvantages, they are easy to implement, but the DC method considers a single factor which resulting in inaccurate results, the BC and CC methods have higher time complexity to collect the shortest path information, the EC method has the limitations in large-scale and heterogeneous networks, and K-shell method utilizes decomposition to determine the node placement throughout the entire network, employing coarse-graining in node differentiation. Consequently, there is a need to develop an influential node identification algorithm that is straightforward to implement, highly accurate, and broadly applicable.

Recently, numerous novel algorithms have been introduced, building upon the enhancements of the classical algorithms mentioned earlier. Most of these algorithms have integrated multiple node attributes. For example, global and local attributes including degree, K-shell, clustering coefficient, and path information were integrated in the recent algorithms^4^, such as PL^15^, ECRM^16^ and GIN^17^ to improve the effectiveness and accuracy of influential node identification. Besides, many algorithms are further improved based on multi-attribute fusion. For example, gravity model-based multi-attribute fusion^18^ identifies the influential nodes by introducing the K-shell, degree, eigenvector centrality or distance between nodes into the gravity model, such as GSM^19^, MCGM^20^ and KSGC^21^. However, since the time complexity of these algorithms generally high, they don't perform well on large-scale networks; Multi-attribute fusion based on EC method uses the relationships between each node and its neighbor nodes to comprehensively estimate, such as PR^22^, Hits^23^ and GSI^24^. Nevertheless, the applicability of these algorithms relies on network structure; Multi-attribute fusion based on entropy centrality^25^ transforms the relationships between nodes and their neighbor nodes into information entropy from the perspective of information dissemination, such as Enrenew^26^, MCDE^27^ and LFIC^28^; Multi-attribute fusion based on voting method, such as VoteRank^29^ and AAVA^8^, is mainly applicable to the scenario of selecting Top-*N* influential nodes. To sum up, it has been a focus of continuous attention in this field to design algorithms with simple implementation, high accuracy, and strong applicability by integrating global and local attributes.

**Figure 1:**
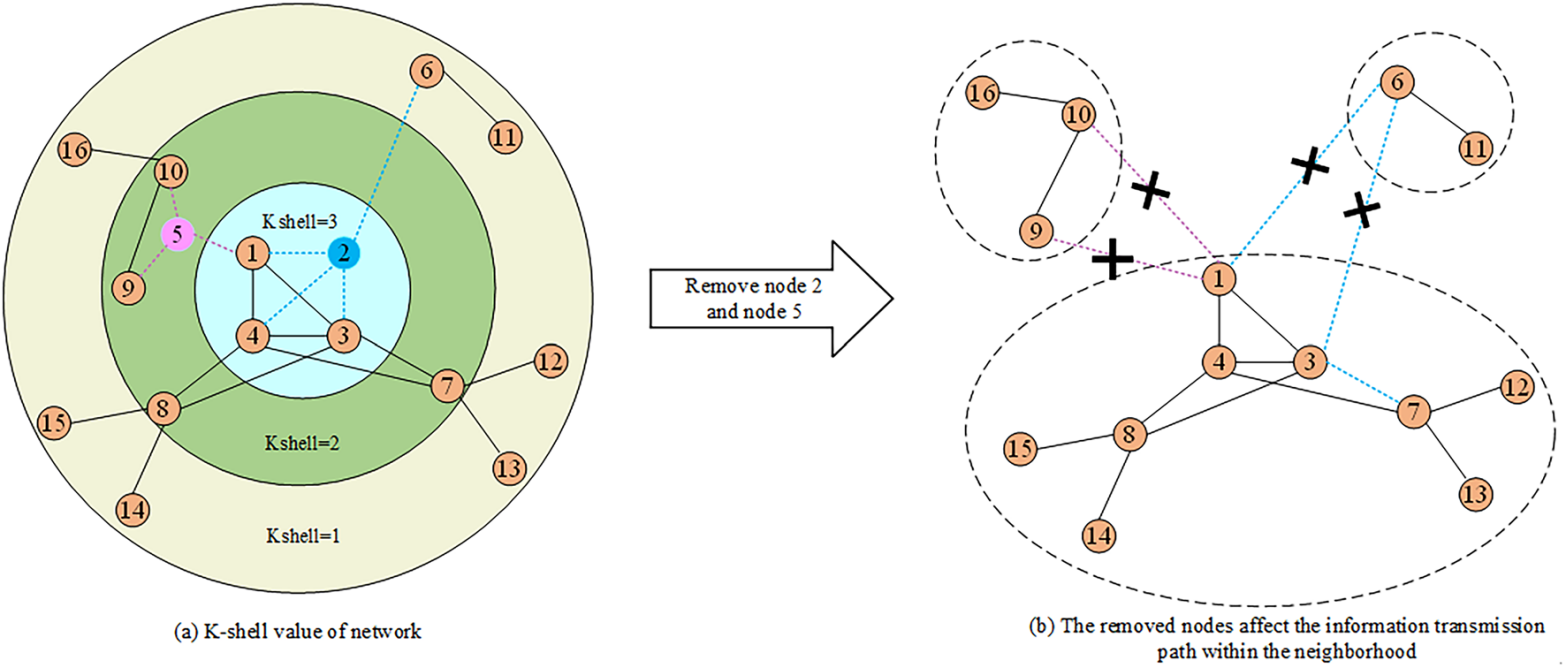
Network model. This example of a complex network comprises 16 nodes and 21 edges. Set nodes 2 and 5 as central nodes and observe the alterations in the paths of information dissemination among their neighbors by removing them.

The majority of the algorithms proposed in the last few years analyze the multiple attributes of nodes when the network structure is fixed and unchanged. However, they ignore that nodes in real networks are usually failure by environmental factors. Hence, we can leverage the concept of network structure robustness^30^ to evaluate the influence of damage on the network and determine the importance of nodes. In practical scenarios, any disruption to the network structure could alter the information dissemination path. During information dissemination process, information will be transmitted between nodes through connections. When a node in the network is removed, the information dissemination paths between nodes within the neighborhood will also be changed. In addition, the influence of failure varies across different nodes. Figure 1 is an example, where node 2 and node 5 are located at different location in the distributed networks, such as federated learning scenarios. As shown in Fig. 1a, the node importance is related to its Kshell value in the whole networks. After node 2 is removed, there’s no path for node 1 and node 3 to reach node 6, similarly, after the removal of node 5, there’s no path for node 9 and node 10 to reach node 1 (as shown in Fig. 1b). The entire network is split into three disconnected sub-networks, that results in some neighbors losing part of the information due to there have no path to transmit information, thus the removed node has the damage influence on the network for information dissemination. Meanwhile, the importance of a node is also related to both its local and global attributes. However, while the local attributes of nodes may be the same, their global attributes could differ. Therefore, when assessing the damage influence of removed node for information dissemination, it is necessary to design more reasonable method to comprehensively consider both the local and global attributes in the network.

Due to the removal of a node will influence the dissemination of information among its neighbors, this paper introduces a novel KLN algorithm, which quantifies the loss of information entropy resulting from the removed node by establishing a KL divergence model within the neighborhood, to estimate the damage influence of the removed node. By amalgamating the damage influence of the one-hop neighbors of the removed node, KLN algorithm provides a more accurate assessment of node importance. The key contributions of KLN algorithm are summarized below:

**(1) Analyzing the damage influence on information dissemination within the neighborhood by removing node.** KLN algorithm utilizes the changes in information dissemination paths among nodes within the neighborhood after node removal to measure the damage influence of the removed node, thereby determines the node importance in the network. This method is more suitable for real-world network scenario.

**(2) Establishing a KL divergence model to evaluate the damage influence of removed node.** Due to KL divergence is used to measure the difference between two probability distributions, thus, the KLN algorithm calculates the difference in probability distributions of information dissemination within the neighborhood before and after removing node, to indicate the damage influence of the removed node. KLN algorithm maps the probability of information dissemination between one-hop neighbors before node removal to an actual probability distribution in KL divergence, and maps the probability of information dissemination between one-hop neighbors after node removal to a fitted distribution. By comparing the K-shell and degree value of the nodes, a KL divergence model is constructed to measure the damage influence of removed node with the neighborhoods comprehensively.

(3) **Assessing the importance of removed node by synthesizing the damage influence of one-hop neighbors.** The importance of the removed node is determined by calculating the damage influence of itself and its one-hop neighbors in the neighborhood. This enhances the accuracy and effectiveness of the importance values of node.

(4) **High performance and scalability.** Experimental results demonstrate that KLN algorithm exhibits high accuracy and is well-suited for networks of different scales. Simultaneously, using epidemic propagation network Epi-2 as a case, this paper identifies and analyzes important nodes within the Epi-2 network, and proposes two strategies for epidemic disease prevention and control.

This paper comprises several sections that discuss the KLN algorithm. Section introduces the relevant methods are detailed. Section "Method description" focuses on the proposed KLN algorithm, with examples analyzed to provide better understanding. Section "Experiment and analysis" assesses the performance of KLN algorithm through experiments conducted on diverse datasets and compares its results with those of other algorithms. Section "Management strategies from case study" conducts a case study on epidemic propagation networks, and proposes epidemic prevention and control strategies based on the finding of key infectious nodes using KLN algorithm.

## Preliminaries

Due to the importance of a node being correlated with both its local and global attributes, we will incorporate the common attributes of degree and K-shell values into the KL divergence model. This will enable us to calculate the loss information entropy to represent the damage influence. These concepts, such as degree, K-shell, Shannon entropy and KL divergence, will be utilized in the development of our proposed algorithm. We will be working within the framework of a network, denoted as *G* = (*V*, *E*).

*Degree Centrality* (DC)^10^: The degree centrality of node *i* is used to measure the number of its neighbors. It’s a direct algorithm to measure the influence of nodes. The degree centrality of a node *i* is calculated:

1 $$DC(i) = \frac{d(i)}{{N - 1}},$$

where *d*(*i*) represents the degree value of node *i*, *N* represents the total number of nodes.

*K-shell decomposition* (KS)^14^ The significance of node placement can be quantified through K-shell decomposition, which evaluates the node importance by breaking down the peripheral layer of nodes into layers of increasing importance towards the innermost layer. The process begins by eliminating nodes with a degree of 1 and their associated edges, followed by a comprehensive traversal of the network until no nodes with 1 degree. These eliminated nodes form a layer with a K-shell value of 1. Subsequently, nodes with a degree of 2 are eliminated, and the algorithm repeats this process for higher degrees. This results in the layer-by-layer decomposition of the network, with nodes in the innermost layer possessing greater importance.

*Shannon entropy*^25^ Shannon entropy represents the index of system uncertainty and is calculated as follows:

2 $$H({\text{x}}) = -\sum\limits_{i = 1}^{N} {p(x_i)} \log p(x_i),$$

where *N* represents the number of possible values of the random variable *x*, *p*(*x*_*i*_) represents the probability function of the random variable *x*_*i*_. When finding influential nodes, their importance can be evaluated by calculating their Shannon information entropy. The entropy value indicates the degree of disorder in the vicinity of the node and the level of uncertainty in transmitting information between the node and others. As uncertainty increases, the amount of information also increases. Therefore, a higher entropy value of a node implies a greater influence.

*Kullback–Leibler divergence* (KL divergence)^31^ The KL divergence quantifies the asymmetry in the difference between two probability distributions, one of which is the real distribution* P*(*x*), while the other is a fitting distribution *Q*(*x*). The KL divergence indicates the loss of information entropy resulting from fitting the real distribution with the theoretical one. It is calculated:

3 $$D_{KL}^{{}} (P(x){||}Q(x)) = \sum\limits_{i = 1}^{N} {P(xi)} \log (\frac{P(xi)}{{Q(xi)}}) = \sum\limits_{i = 1}^{N} {P(xi)} \log P(xi) - \sum\limits_{i = 1}^{N} {P(xi)} \log Q(xi),$$

where *P*(*x*_*i*_) is the probability of the *i*th element in *P*(*x*), *Q*(*x*_*i*_) is the probability of the *i*th element in the probability distribution *Q*(*x*) of the event fitted by the theory. The larger value of KL divergence proves that the fitted probability distribution loses more information entropy^32,33^.

By synthesizing the degree, K-shell attributes of complex networks in the KL divergence model, this paper proposes an algorithm for finding influential nodes based on Kullback–Leibler divergence model within the neighborhood (KLN).

## Method description

### Algorithm proposal

Assume a network $G = (V,E)$ with *V* nodes and *E* edges, and *Γ*(*i*) represents the neighbors set of node *i*.

#### Definition 1

(*Path Change Factor*) When node *i* in the network is removed, the shortest distance between its neighbors may be changed. The probability of information dissemination is related to the distance between the two nodes. The shorter distance between two nodes, the higher probability of successful information dissemination, thus, after the removal of node *i*, we define the calculation of the path change factor of between its neighbor nodes *n* and *j* as:

4 $$\lambda (n,j) = \frac{1}{{l_{{}}^{{}} (n,j)}}.$$

where *l*(*n*, *j*) represents the shortest distance between nodes* n* and* j*. If there’s still a path between node *n* and *j* after the removal of node *i*, the path change factor is equal to the reciprocal of the shortest distance *l*(*n*, *j*). When there is no path for information dissemination after the removal of node *i*, the path change factor is set to 1/2. This is because, before the removal of node *i*, nodes *n* and *j* transmit information through node *i* by 2 hops between them.

#### Definition 2

(*Real probability distribution of information dissemination*) The real probability distribution *P*_*i*_(*x*) of information dissemination can be determined based on the neighbors of node *i*. When node *i* is operational, it enables its neighbors to transmit information to other neighbors. Therefore, the real probability distribution of information dissemination *P*_*i*_(*x*) can be defined as:

5 $$P_{i}^{{}} (x) = \left\{ {p_{i}^{{}} (1),p_{i}^{{}} (2),\ldots p_{i}^{{}} (j),\ldots p_{i}^{{}} (n)} \right\},$$

where *p*_*i*_(*n*) is the *n*th element in *P*_*i*_(*x*), {1, 2, 3,…*j*,.. *n*} represents the neighbors of node *i*, *p*_*i*_(*j*) represents the real probability of information dissemination for neighboring node *j.* A higher degree of node *d*(*j*) will increase the probability of information dissemination to other nodes, thus, *p*_*i*_(*j*) is defined as:

6 $$p_{i}^{{}} (j) = \frac{d(j)}{{\sum\nolimits_{j \in \Gamma (i)} {d(j)} }},$$

#### Definition 3

(*Fitting probability distribution of information dissemination*) After the removal of node *i* and its connecting edges, the probability of information dissemination between each neighbor will be changed within the neighborhood. The fitting probability distribution *Q*_*i*_(*x*) of information dissemination between all neighbors of node *i* is defined as:

7 $$Q_{i}^{{}} (x) = \left\{ { q_{i1}^{{}} (x),q_{i2}^{{}} (x),q_{i3}^{{}} (x),\ldots q_{ij}^{{}} (x),\ldots q_{in}^{{}} (x)} \right\}$$

where *q*_*i*_(*n*) is the *n*th element in *Q*_*i*_(*x*), {1, 2, 3,…*j*, *n*} represents the neighbors of node *i*, *q*_*ij*_(*x*) represents the fitting probability distribution of information dissemination of the neighboring node *j* with all the other neighbor of node *i*, which is defined as:

8 $$\begin{gathered} q_{i1}^{{}} (x) = \left\{ {\chi_{i1}^{{}} (1), \chi_{i1}^{{}} (2), \ldots \chi_{i1}^{{}} (j),\chi_{i1}^{{}} (n)} \right\} \hfill \\ q_{i2}^{{}} (x) = \left\{ {\chi_{i2}^{{}} (1),\chi_{i2}^{{}} (2),\ldots \chi_{i2}^{{}} (j),\chi_{i2}^{{}} (n)} \right\} \hfill \\ \ldots \hfill \\ q_{ij}^{{}} (x) = \left\{ {\chi_{ij}^{{}} (1),\chi_{ij}^{{}} (2),\ldots \chi_{ij}^{{}} (j),\chi_{ij}^{{}} (n)} \right\} \hfill \\ \ldots \hfill \\ q_{in}^{{}} (x) = \left\{ {\chi_{in}^{{}} (1),\chi_{in}^{{}} (2),\ldots \chi_{in}^{{}} (j),\chi_{in}^{{}} (n)} \right\}, \hfill \\ \end{gathered}$$

with

9 $$\chi_{in}^{{}} (j) = \frac{{\sqrt {\lambda (n,j)} {*}d^{\prime}(j)}}{{\sum\nolimits_{j \in \Gamma i} {\sqrt {\lambda (n,j)} *d^{\prime}(j)} }},$$

where $\chi_{{{\text{in}}}} (j)$ represents the fitting probability distribution of information dissemination between the neighbor nodes *n* and* j* after node *i* is removed, its calculation is related to the shortest distance and degree value between nodes, $\lambda (n,j)$ represents the path change factor of the information dissemination between nodes *n* and *j* calculated by Definition 1, and d*′*(*j*) represents the current degree of node* j* after node *i* is removed.

#### Definition 4

(*KL divergence model*) By using the real probability distribution *P*_*i*_(*x*) and the fitting probability distribution *q*_*ij*_(*x*) between node *j* and other neighbors after the node *i* is removed, the KL divergence *D’*_*ij*_(*P*||*Q*) can be calculated by Eq. (10), which represents the lost information entropy when transmitting information through node *j* after node *i* is removed. By integrating the Kshell value of all the one-hop nodes within the neighborhood of node *i*, the KL divergence *D*_*i*_(*P*||*Q*) of node *i* can be calculated in Eq. (11):

10 $$D_{ij}{\prime} (P||Q) = \sum {_{x \in X}^{{}} Pi({\text{x}}){*}\log \frac{Pi(x)}{{q_{ij}^{{}} (x)}}},$$

11 $$D_{i}^{{}} (P||Q) = \sum\limits_{j = 1}^{n} {_{{j \in \Gamma_{i}^{{}} }}^{{}} Kshell(j){*}D_{ij}{\prime} (P||Q)},$$

#### Definition 5

(*Damage influence*) The damage influence of removed node *i* is related to its KL divergence value and its degree *d*(*i*), which is defined as:

12 $$DE(i){ = }D_{i}^{{}} (P||Q) + d{(}i{)},$$

#### Definition 6

*(Node importance*) The importance of the removed node is determined by assessing the damage influence of the node itself and its one-hop nodes in neighborhood. Taking node *i* as the center node, the damage influence values of all the one-hop nodes in its neighborhood are calculated, and the importance value *KLN*(*i*) of node *i* is finally obtained as:

13 $$KLN{(}i{) = }DE(i) + \sqrt {\sum\nolimits_{j \in \Gamma i} {DE(j)} },$$

### Algorithm execution and example

**Figure 2:**
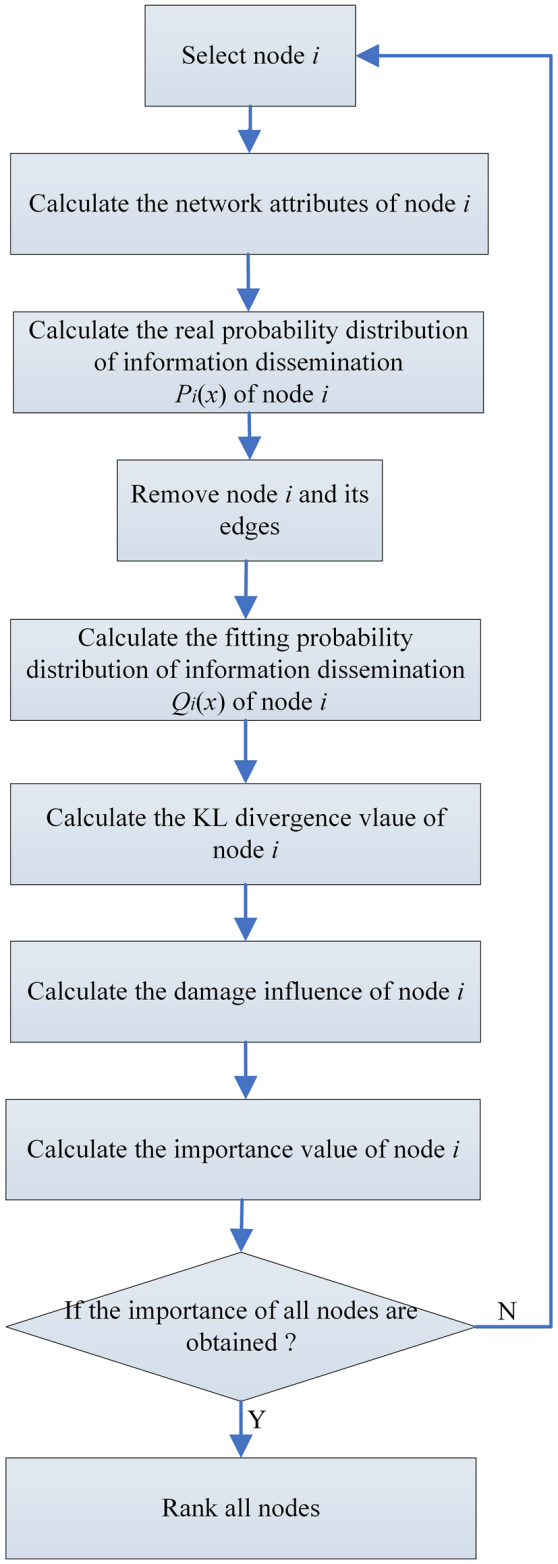
Flow chart of KLN. According to the principles of the KLN algorithm, the algorithm's execution process can be divided into six steps.

After initializing the network, a node *i* to be removed is first selected. The execution of KLN is divided into 6 steps. Step 1: Calculate the global and local attributes of node *i*. Step 2: Calculate the real probability distribution *P*_*i*_(*x*) of information dissemination of one-hop neighbors around node *i*. Step 3: After removing node *i*, calculate the fitting probability distribution *Q*_*i*_(*x*) of all the neighbors of node *i*; Step 4: Calculate the KL divergence value of node *i*. Step 5: Calculate the damage influence of node *i*, and after the calculation is completed, traverse the network successively until the damage influence values of all nodes in the network are obtained. Step 6: Calculate the importance ranking results of all the nodes. Step 7: Rank all nodes in the network. The preceding steps are performed as shown in Fig. 2.

**Figure 3:**
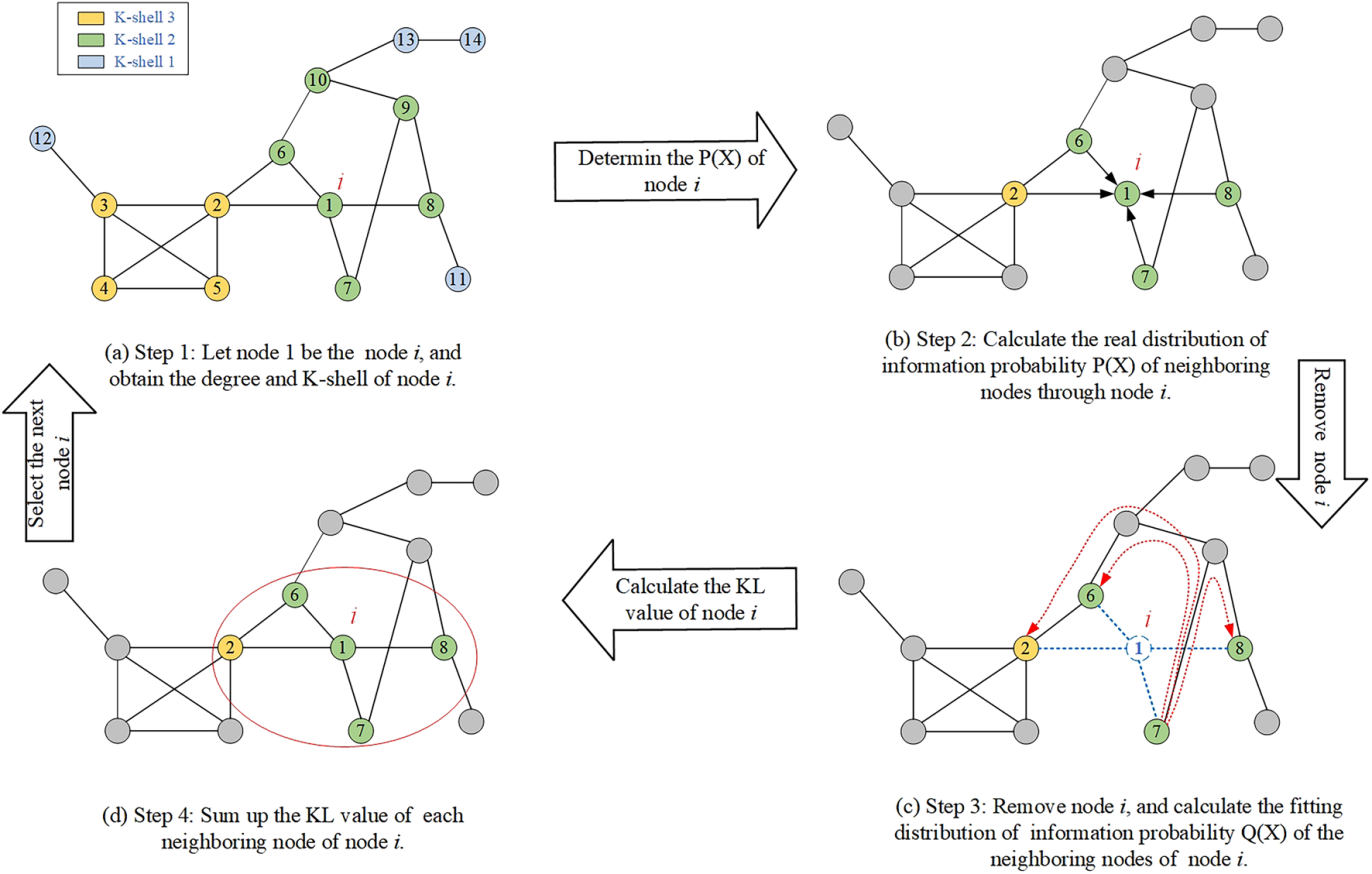
Solving process of KL divergence in the algorithm. In this example complex network, there are 14 nodes and 18 edges, the maximum K-shell value is 3. Setting node 1 as the central node, and removing it, we analyze the changes in the paths among the surrounding nodes after the removal of node 1 to obtain the KL divergence value. This process is then iteratively repeated by selecting different central nodes until all nodes have their respective KL divergence values. Additional, combining the K-shell and degree attributes of the nodes, we calculate the damage influence for each central node.

We will give an example of the execution process of KLN algorithm in Fig. 3. The removal node *i* for the network is designated as node 1, and the algorithm's execution process is demonstrated with examples at each step.

Step 1: Calculate the local and global network attributes. Calculate the degree, K-shell values of node 1*.* In Fig. 3a. The degree value of node 1 is 4, its K-shell value is 2, and its neighbors are the nodes 2, 6, 7, and 8.

Step 2: Calculate the *P*(*x*) of node 1. According to the connection relation of information dissemination between nodes. In Fig. 3b, all the four neighbors of node 1 can transmit information through node 1. According to Eq. (5) and (6), the final probability distribution *P*_1_(*x*) of information dissemination between the neighbors is calculated as:

14 $$\begin{gathered} P_{1}^{{}} (x) = \left\{ {p_{1}^{{}} (2),p_{1}^{{}} (6),p_{1}^{{}} (7),p_{1}^{{}} (8)} \right\} \hfill \\ = \left\{ {0.384,0.231,0.154,0.231} \right\}. \hfill \\ \end{gathered}$$

Step 3: Calculate the *Q*(*x*) of neighbors. Remove node 1 and its connection edges. Take the node 7, a neighbor of node 1, as an example. In Fig. 3c, when the information is transmitted between node 7 and other neighbors 2, 6, 7, and 8, the first step is to determine the shortest distance to 2, 6, 7 and 8, which equals to 4, 3, 1and 2, respectively. Therefore, using Eq. (8) and (9), the following can be obtained:

15 $$\begin{gathered} q_{17}^{{}} (x) = \left\{ {\chi_{17}^{{}} (2),\chi_{17}^{{}} (6),\chi_{17}^{{}} (7),\chi_{17}^{{}} (8)} \right\} \hfill \\ = \left\{ {0.359,0.207,0.180,0.254} \right\}, \hfill \\ \end{gathered}$$

16 $$\chi_{17}^{{}} (2) = \frac{{\sqrt{\frac{1}{4}} {*4}}}{{\sqrt{\frac{1}{4}} {*4} + \sqrt{\frac{1}{3}} {*3} + 1{*1} + \sqrt{\frac{1}{2}} {*2}}} \approx 0.359,$$

The information dissemination probability between all neighbors of node 1 and other neighbors is calculated successively, and the final distribution of *Q*_1_(*x*) in Eq. (7), is finally obtained as follows:

17 $$Q_{1}^{{}} (x) = \left\{ { q_{12}^{{}} (x),q_{16}^{{}} (x),q_{17}^{{}} (x),q_{18}^{{}} (x)} \right\} = \left[ \begin{gathered} 0.533,0.267,0.067,0.133 \hfill \\ 0.517,0.259,0.075,0.149 \hfill \\ 0.359,0.207,0.180,0.254 \hfill \\ 0.341,0.197,0.121,0.341, \hfill \\ \end{gathered} \right]$$

Step 4: Calculate the KL divergence value of the node 1. According to Eq. (11), the fitting distribution *q*_*in*_(*x*) of each neighbor node in *P*_1_(*x*) and *Q*_1_(*x*) is calculated respectively to obtain the lost information entropy *D*_*ij*_’(*P*||*Q*) due to the removal of node 1. According to the K-shell attribute of neighbor node, using Eq. (11), the KL divergence of node 1 is finally obtained:

18 $$\begin{gathered} D_{1}^{{}} (P||Q) = Kshell(2){*}D_{12}{\prime} (P||Q) + Kshell(6){*}D_{16}{\prime} (P||Q) + \hfill \\ Kshell(7){*}D_{17}{\prime} (P||Q) + Kshell(8){*}D_{18}{\prime} (P||Q) \hfill \\ = 3{*0}{\text{.042}} + 2{*}0.031 + 2{*}0.002 + 2{*}0.013 = 0.218. \hfill \\ \end{gathered}$$

Step 5: Calculate the damage influence of node 1. In Fig. 3d, the degree of node 1 is equal to 4. According to Eq. (13), the final damage influence of node 1 is *DE*(1) = 0.218 + 4 = 4.218.

**Figure 4:**
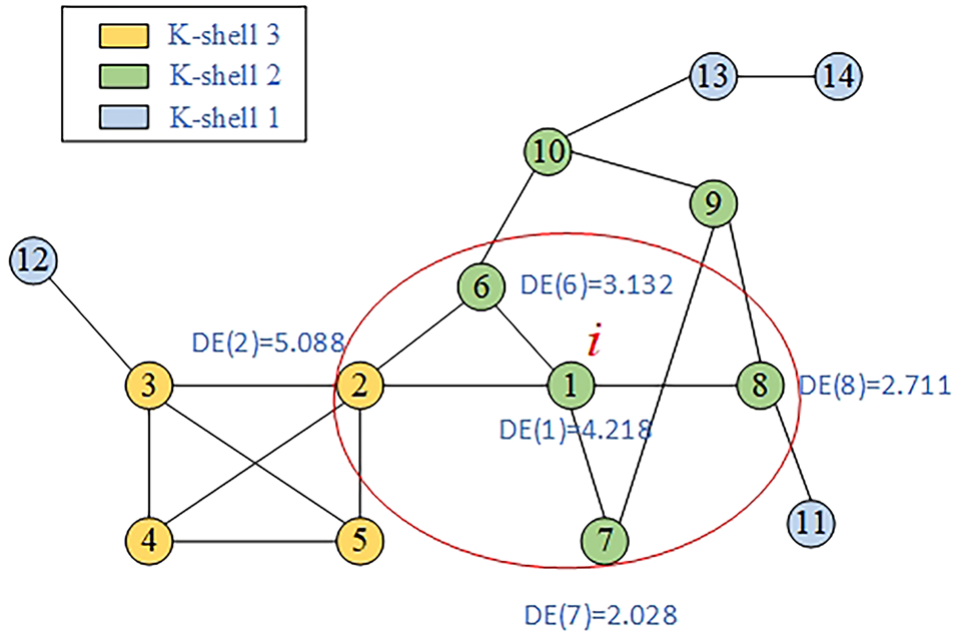
Example of node importance. By quantifying the damage influence in neighborhood centered around node 1, which includes all one-hop neighbors (2, 6, 7, 8), along with the damage influence on node 1 itself, we obtain the importance value of node 1.

Step 6: Calculate the final importance value of node 1. After the damage influence values of all the nodes are calculated successively, as shown in Fig. 4, the damage influence of node 1 and its one-hop neighbors 2, 6, 7, and 8 are integrated in neighborhood.

According to Eq. (13), the final importance value of node 1 is equal to:

19 $$KLN{(}i{) = }4.{218} + \sqrt {5.088 + 3.132 + 2.028 + 2.711} = 7.826$$

Step 7:Rank all nodes. The importance ranking results and values in this example network are in Table 1.

**Table 1:** Ranking results and values in toy network. Using the calculations of the KLN algorithm, it is determined that node 2 has the greatest influence in the network. Based on the sorting of node numbers, the importance of nodes decreases progressively from left to right.

Node	2	1	3	6	4	5	9	10	8	7	13	12	11	14
Value	9.22	7.83	7.15	6.65	6.44	6.44	5.90	5.89	5.65	4.73	3.74	2.92	2.66	2.31

From Table 1, we can observe that among these 14 nodes, node 2 has the highest importance value. Among nodes with the same color, their K-shell values are identical. KLN algorithm can effectively differentiate the importance values of these nodes with identical k-shell values. Only nodes 4 and 5 have identical importance values because their local attributes, global attributes, and neighbors are the same in the network. Therefore, through the KLN algorithm, it is possible to effectively distinguish the importance values of different nodes. The accuracy and effectiveness of node importance values will be further validated in detail in the experiments of the Section "Experiment and analysis".

### Algorithm complexity

The implementation process of KLN is as follows.
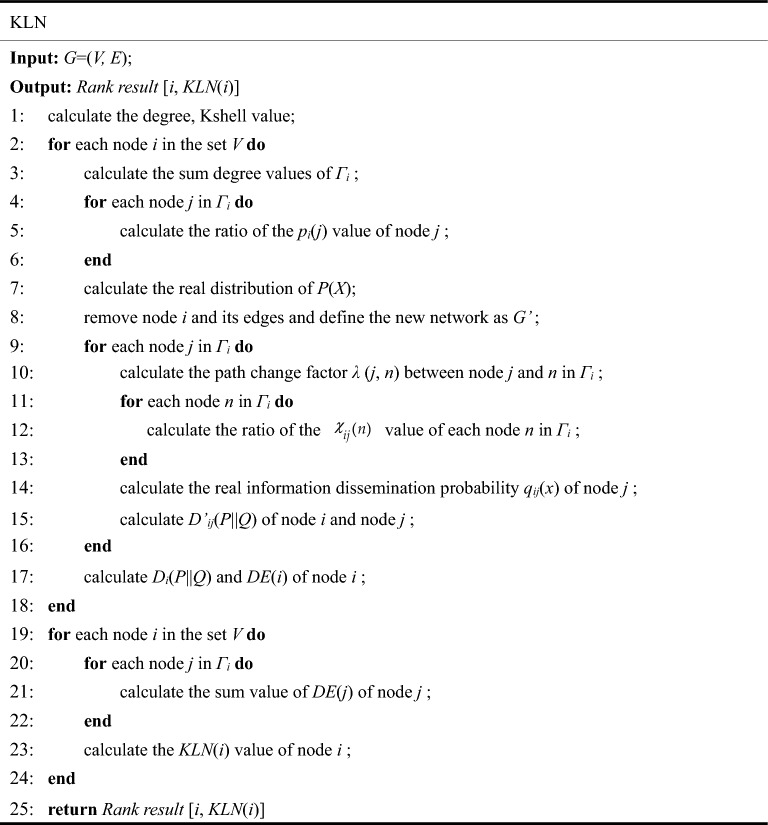


The first line of the execution code for this algorithm calculates the degree and Kshell value of each node, whose time complexity is *O*(< *k* > *n*), and < *k* > and *n* represents the average degree and nodes number, respectively. In 2–19 lines of the execution code calculates the KL divergence* D*_*i*_(*P*||*Q*) and damage influence *DE*(*i*) value of each node, the external loops number are *n*. The first inner loop calculates the *P*(*X*) probability before node removal, with the number of loops equal to < *k* > . The second inner loop, which determines *λ*(*n*, *j*), the distribution of *q*_*ij*_(*x*), and the KL divergence value *D*’_*ij*_(*P*||*Q*) of the nodes after removal, has < *k* > loops, resulting in an overall time complexity of *O*(< *k* > ^*2*^*n*). In lines 20–26, the external loops are again *n*, and the inner loop calculates the final influence *KLN*(*i*) of each node, whose time complexity is *O*(< *k* > *n*). In summary, the proposed KLN has a whole-time complexity of *O*(< *k* > ^*2*^*n*).

## Experiment and analysis

The experiments are run on a Win10 systems, i3-10100 CPU, and 8 GB memory of the computer. Ten representative real network data are selected in this experiment, and the KLN is compared with some early classical algorithms such as DC, KS(K-shell), EC, PR, and the recently proposed methods such as PL, Enrenew, MCDE, ECRM, GSM, GSI, and MCGM. As a result, the proposed algorithm’s performance is evaluated by analyzing the experimental results. We will use Top-10 nodes, Kendall *τ* the infection capacity of all network nodes and the Top-10 nodes to validate the accuracy and effectiveness of the identified critical nodes.

The types of ten datasets are: social networks include Karate, Dolphins, and Friendships, interactive networks include Football, transport networks include Euroroad and Usair, protein networks include Protein, electric power networks include Powergrid, paper citation networks include HepPh, and collaborative networks include Ca-astroph. Table 2 shows the relevant features of these real networks.

**Table 2:** Network characteristics. In these datasets, *|E|*, *|V|*, *d*_*max*_, <*c*>, <*k*> separately refers to the edges number, nodes number, max degree, clustering coefficient, average path length in the datasets.

DataSets	|E|	|V|	d_max_	<c>	<*k*>
Karate	78	34	17	0.588	2.408
Dolphins	159	62	12	0.303	3.357
Football	616	115	12	0.403	2.508
Euroroad	1417	1174	5	0.02	18.32
USair	2408	1226	34	0.073	5.929
Friendships	12,534	1858	272	0.167	3.453
Protein	2277	1870	56	0.171	6.81
Powergrid	6594	4941	19	0.107	18.99
HepPh	118,521	12,008	491	0.699	4.673
Ca-astroPh	198,050	18,771	236	0.677	4.194

### Top-10 nodes

Among the ten networks, Dolphins, Euroroad, Protein, and Powergrid are selected from the networks with different node quantity ranges. To analyze the accuracy of the algorithms, we compared the Top-10 nodes of the sorting results across the 12 algorithms. As shown in Tables 3, 4 and 5, ten nodes are represented by different colors in KLN. If the nodes identified by KLN also appear in the Top-10 nodes of other compared algorithms, they are marked with the same color. If the nodes of the same color and the result of KLN have the same sorting order, then the node is represented by an underscore.

Table 3 shows that in Dolphins network, the Top-10 nodes sorted by KLN algorithm all appear in the other 11 algorithms, in which KLN has the same 10 nodes selected by DC, the same 9 nodes as MCDE, the same 8 nodes as MCGM, GSI, ECRM, Enrenew, EC, PL and PR, and the same 5 nodes and 3 nodes as GSM and K-shell, respectively. In addition, in terms of node sorting, KLN has the highest similarity with MCGM, with 5 nodes in the same order. Therefore, the KLN algorithm performs better in Dolphins network.

**Table 3:**
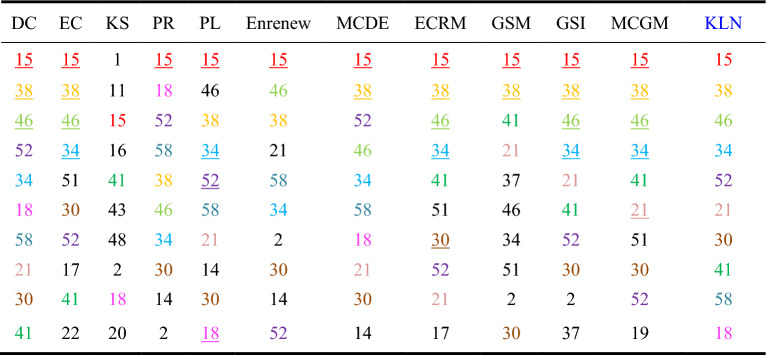
Top-10 nodes in Dolphins. Colored nodes in each compared algorithm represent the Top-10 nodes identified by the KLN algorithm, and the underline nodes indicate that the order of these nodes is consistent with the order determined by the KLN algorithm.

Table 4 shows that in Euroroad network, KLN has the same 9 nodes selected by DC, PL, Enrenew, MCDE, and GSI, the same 8 nodes as the ECRM, the same 6 nodes as EC and PR, the same 5 nodes and 2 nodes as MCGM and GSM, respectively, and the same 1 node as K-shell. This is because of the low discrimination of nodes caused by the coarse granulation of K-shell algorithm. Additionally, in the consistency of node sorting, KLN has the highest similarity with GSI, with 4 nodes in the same order. Therefore, the KLN algorithm performs better in Euroroad network.

**Table 4:**
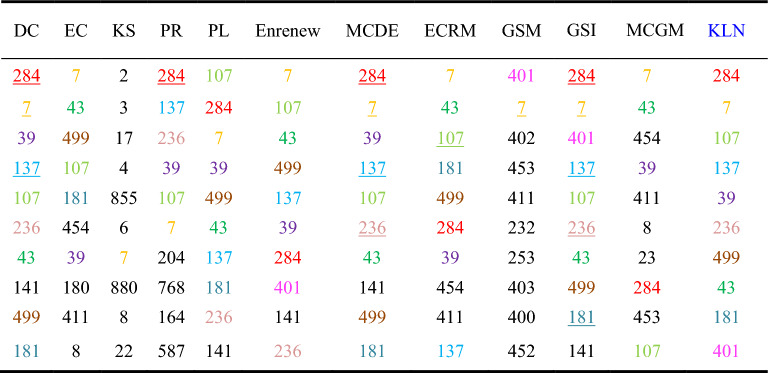
Top-10 nodes in Euroroad. Colored nodes in each compared algorithm represent the Top-10 nodes identified by the KLN algorithm, and the underline nodes indicate that the order of these nodes is consistent with the order determined by the KLN algorithm.

Table 5 shows that in Powergrid network, KLN has the same 8 nodes selected by MCDE, Enrenew, and PL, the same 6 nodes as the ECRM, the same 5 nodes as MCGM, GSI, EC, and DC, and the same 4, 3 and 2 nodes as GSM, K-shell, and PR, respectively. Besides, in terms of node sorting, KLN has the highest similarity with GSI and MCDE, with 2 nodes in the same order. Therefore, the KLN algorithm performs better in Powergrid network.

**Table 5:**
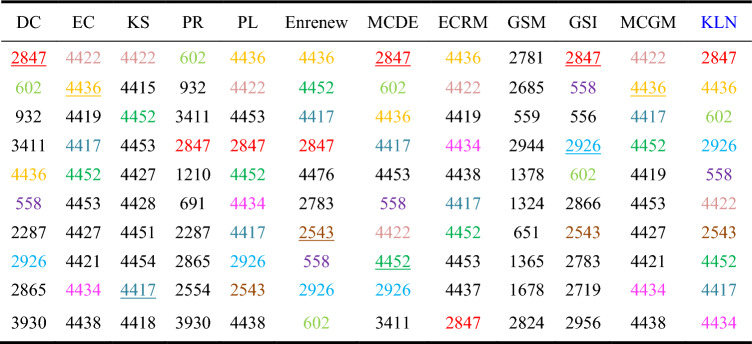
Top-10 nodes in Powegrid. Colored nodes in each compared algorithm represent the Top-10 nodes identified by the KLN algorithm, and the underline nodes indicate that the order of these nodes is consistent with the order determined by the KLN algorithm.

Table 6 shows that in HepPh network, KLN has the same 10 nodes selected by MCGM and PL, the same 9 nodes as GSI, EC, and ECRM, the same 8 nodes as DC, MCDE, and GSM, the same 7 nodes as Enrenew, the same 5 nodes as PR, and the same 2 nodes as K-shell. Moreover, in terms of node sorting, KLN has the highest similarity with MCGM, with 8 nodes in the same order. Therefore, the KLN algorithm performs better in HepPh network.

**Table 6:**
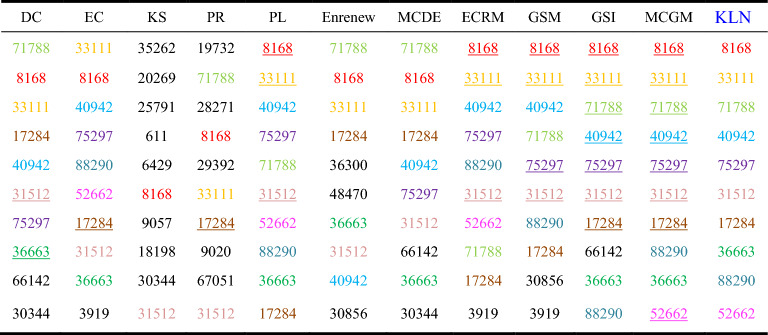
Top-10 nodes in HepPh. Colored nodes in each compared algorithm represent the Top-10 nodes identified by the KLN algorithm, and the underline nodes indicate that the order of these nodes is consistent with the order determined by the KLN algorithm.

### Kendall τ based on SIR model

In this section, Kendall *τ* value^34^ is used to describe the algorithm’s execution results and the accuracy of the sorting results under SIR model. SIR model is a widely utilized infectious disease model^35^. It is well-suited to accurately simulate the progression of infections between nodes. This model includes susceptible (*S*) node, infective (*I*) node and recovery (*R*) node. In each period, when *S* and *I* nodes get in contact, the *S* node becomes an *I* node with an infection probability of *α*. At the same time, an *I* node becomes a *R* node with a cure rate of *β*, and the *R* nodes in the infection process has ceased. In accordance with the SIR model's infection relationship, a number of nodes in network have been designated as *S* nodes with a specific initial probability. The number of *I* node in a period is calculated to represent the infection capacity of nodes.

**Figure 5:**
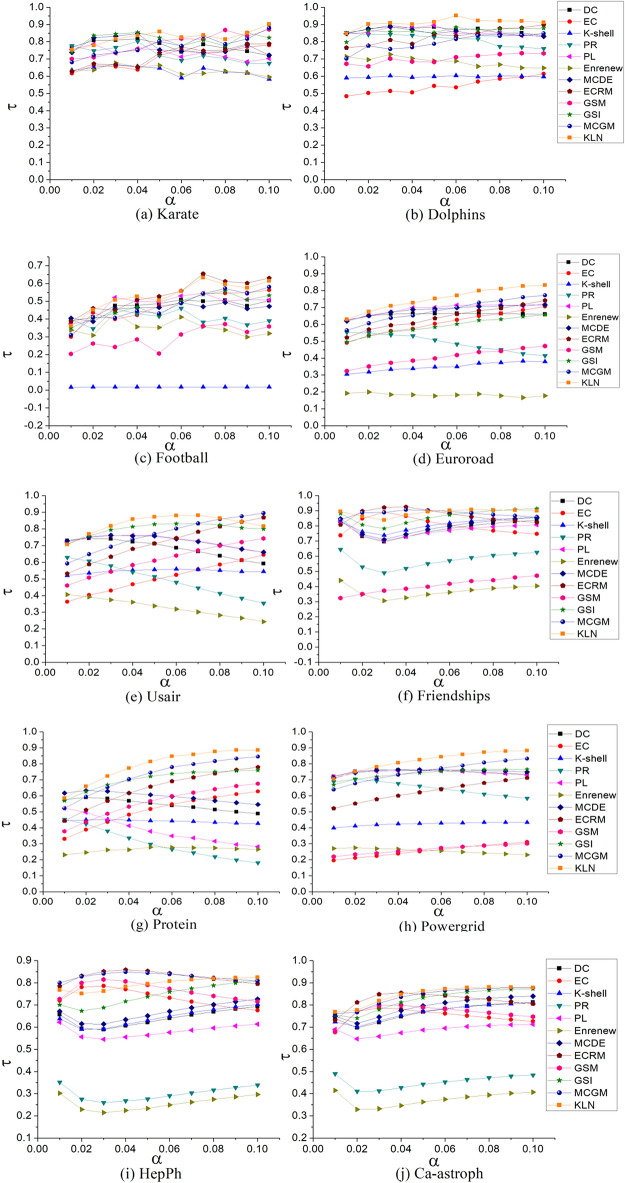
Comparison of Kendall *τ* results in 10 complex networks. In this experiment, infection probability *α* is set as [0.01–0.1], to avoid the value of α being too large or too small. A too-large value of *α* may lead to too-fast infection speed, so the importance of a single node cannot be effectively evaluated. Based on SIR model, the final Kendall *τ* values of 12 algorithms in different colors are the average value obtained after 1000 iterations.

After executing the SIR model, we analyzed the sorting results of both the proposed and comparison algorithms, and conducted correlation analysis with the SIR results. The accuracy of the algorithm results was reflected using the Kendall *τ* values. A larger value indicates greater similarity between the algorithm's sorting result and that of SIR Model, and therefore higher accuracy. The formula for Kendall *τ* is defined as follows:

20 $$\tau (X,Y) = \frac{a - b}{{0.5n(n - 1)}},$$

where *τ*(*X*,*Y*) is utilized to estimate the similarity of elements between sequences *X* and *Y*. It considers the total number of elements *n* along with the consistent *a* and inconsistent *b* pairs in both sequences *X* and *Y*. In Fig. 5, we present the comparative Kendall *τ* values between the sorting results of the 12 algorithms across 10 different networks.

As shown in Fig. 5, KLN has the best overall effect in the Dolphins, Euroroad, Protein, Powergrid and Ca-astroph networks. In Karate network, in the range of *α* = [0.05–0.07] and [0.09–0.1], the Kendall *τ* value of KLN is the largest. In the range of *α* = (0.01–0.04), the initial probability of KLN is lower than that of DC and GSI, whereas near *α* = 0.08, EC has the best effect. In Football network, KLN and ECRM are at higher levels, PL has the largest *τ* value when *α* = 0.03, and K-shell has the lowest value in this network. This is because the network structure is single, the node differentiation is decreased due to some nodes have the same K-shell values. In Usair network, in the range of* α* = [0.02–0.08], the τ value of KLN is at the highest level. With the increase in *α,* the effect of KLN is merely lower than that of MCGM and ECRM. In Friendships network, in the range of *α* = [0.02–0.04], the performances of MCGM, GSI, and ECRM are exceptional, and in other ranges, the Kendall *τ* of KLN is at a high level. In HepPh network, the Kendall *τ* value of KLN is the largest in the range of *α* > 0.8, while it is overall lower than that of ECRM and MCGM in the other range. To sum up, in the 10 networks, the Kendall *τ* value of KLN is overall optimal in 5 networks (Dolphins, Euroroad, Protein, Powergrid and Ca-astroph networks), and in part of the ranges of the remaining 5 networks, it also has a large Kendall *τ* value. Therefore, KLN has high accuracy in influential nodes identified by the SIR model.

### Infection capacity

**Figure 6:**
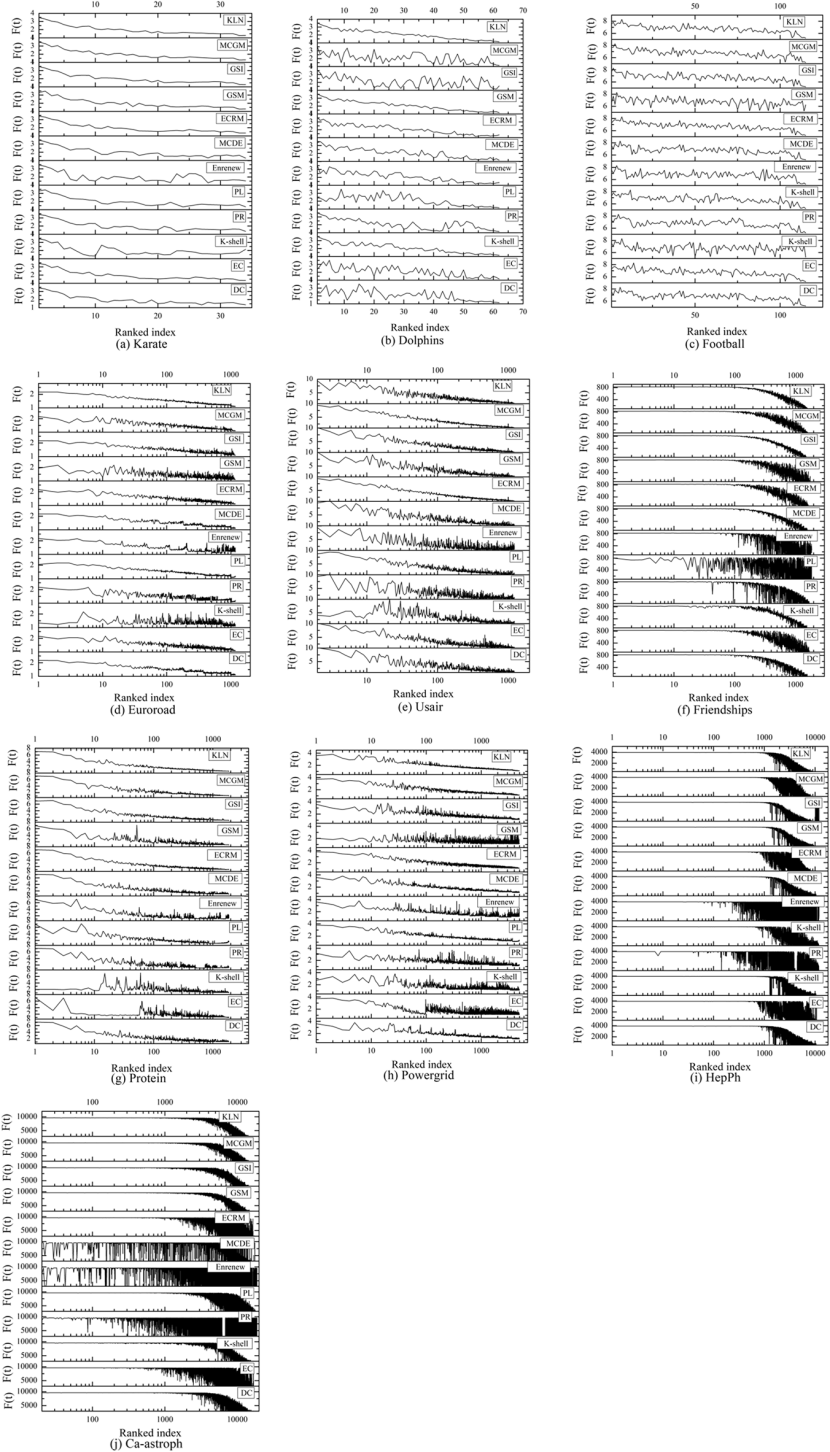
Comparison of node infection capacity in 10 networks. In the experiment, *α* = 0.1, *β* = 1, and the iterations is set to 1000 (except 100 iterations for Ca-astroph network). According to the scale of network, the curve data is displayed linearly for the first 3 networks, while it is displayed in log10 scale for the remaining networks.

This section analyzes the infection capacity of nodes in the entire network. The node sorting distribution in the result of each algorithm is mapped to the sorting result of SIR model, and the nodes number corresponding to infected nodes in SIR model is plotted as a curve. With the decrease in node importance order, the ideal curve should show a smooth downward trend. It is proved that the sorting result of KLN is more accurate, and the infection capacity of this kind of sorting result is stronger in the network. Figure 6 shows the infection capacity curves of 12 algorithms in 10 networks.

As shown in Fig. 6, the curve of KLN shows a smooth downward trend and has fewer burrs of the curves in seven networks: Karate, Dolphins, Euroroad, Friendships, Powergrid, HepPh, and Ca-astroph, thus the whole infection effects have the better performance. In Football network, the curve change difference of all algorithms is small, which is caused by the large degree value difference of nodes in Football, thus the overall infection effects of 12 algorithms are similar. In Usair network, the overall effects of KLN, ECRM, and GSI are almost the same, there is no significant difference in the smoothness of three curves, and the infection effect is obviously better than other algorithms. In Protein network, the ECRM has the smoothest curve, so it has the best overall infection effect, but the overall trend of the Top-10 nodes of the KLN is basically the same as that of ECRM. In summary, the infection effect of KLN in most networks exhibits a smooth downward trend and fewer burrs. This indicates that this algorithm has a good overall effect when its sorting results are used to perform network infection.

### Infection capacity of Top-10 nodes

To thoroughly evaluate the algorithm's effectiveness, we select the Top-10 nodes among the 12 algorithms and use them to infect surrounding nodes. For this purpose, we set *α* and *β* to 0.01 and 0.1, respectively, and keep track of the number of infected nodes after each round. To ensure statistical significance, we conduct 1000 iterations over 30 rounds and compute the average number of infected nodes.

**Figure 7:**
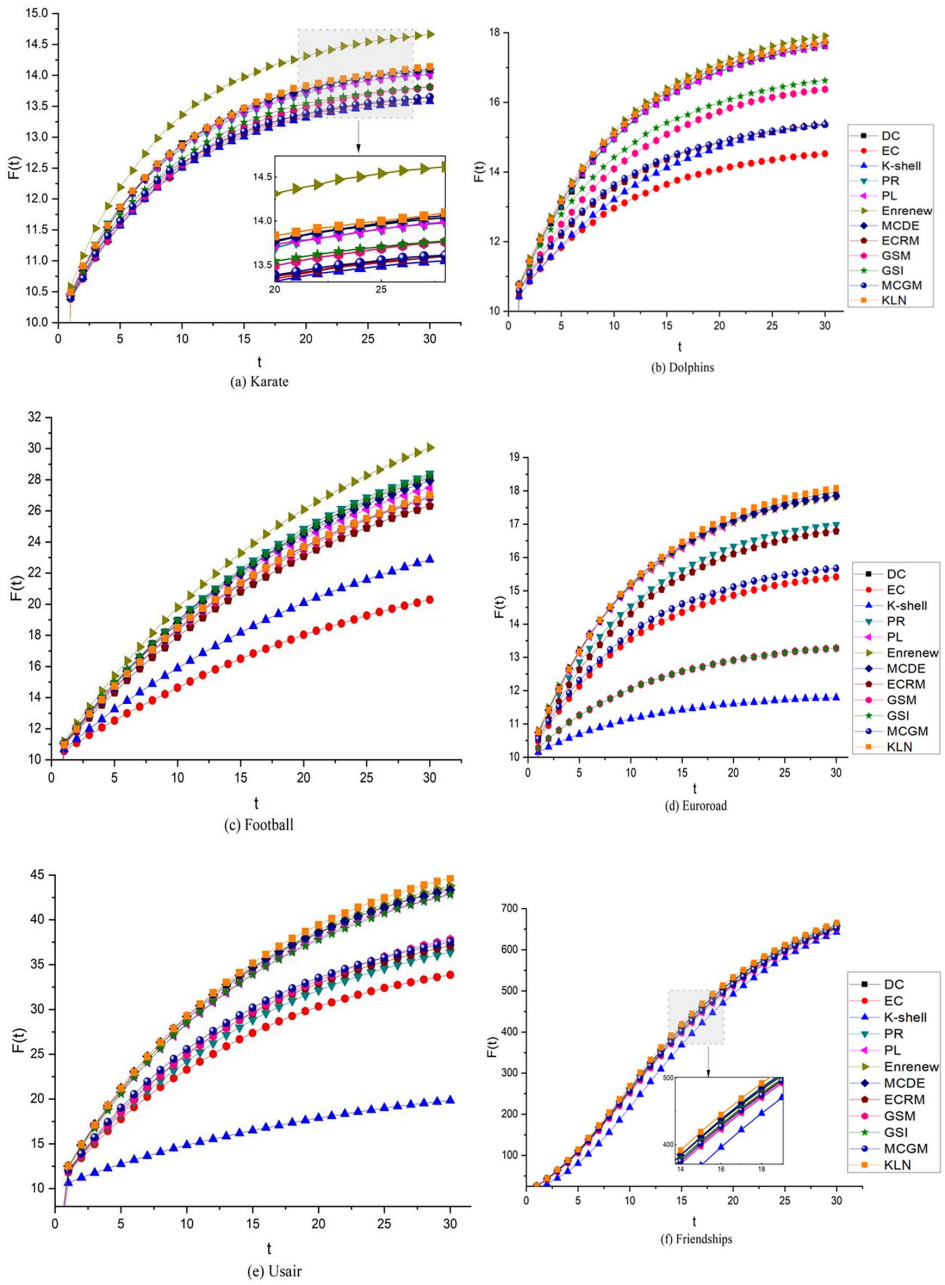

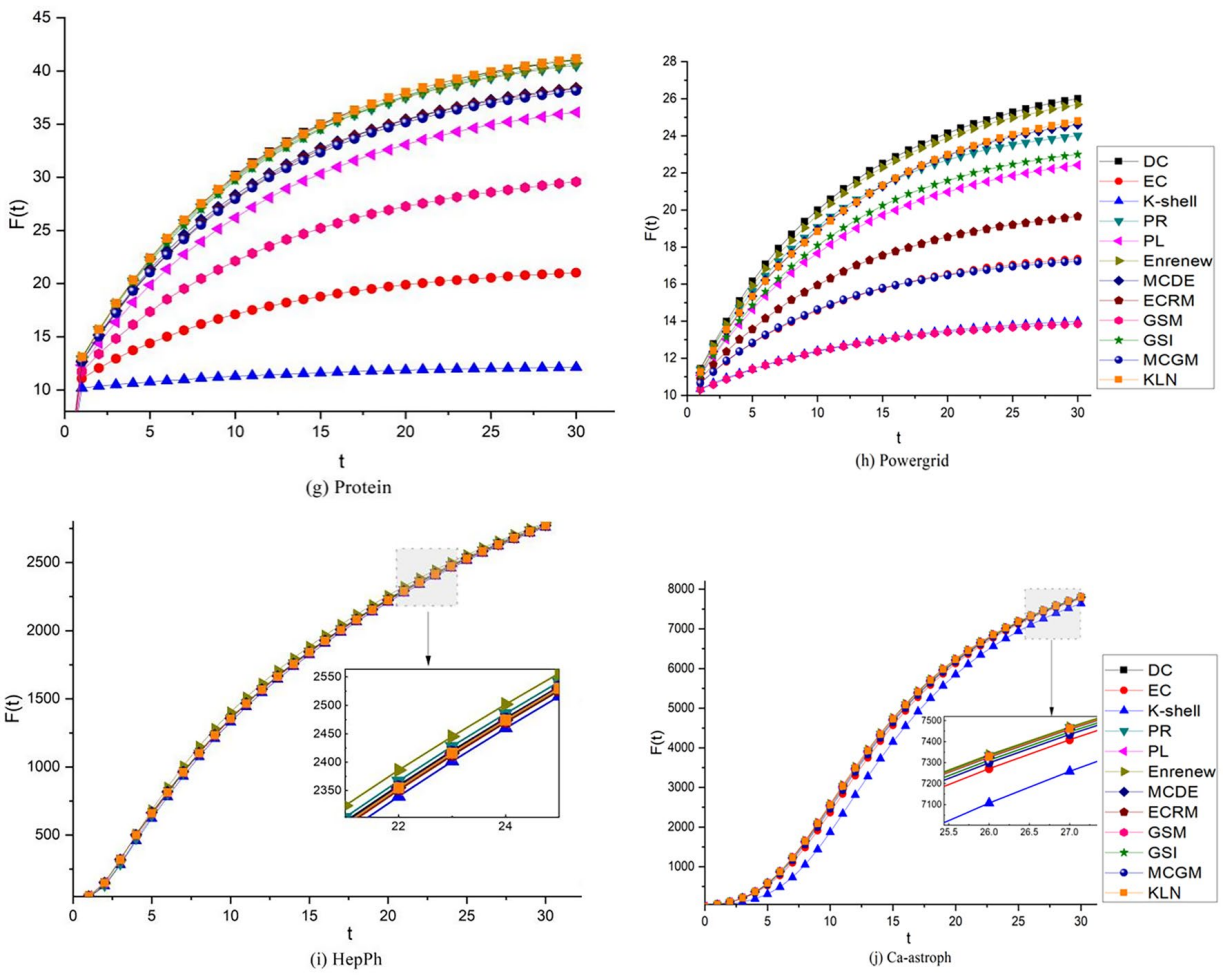
Comparison of the Top-10 nodes infection capacity in 10 complex networks. In the experiment, *α* = 0.1, *β* = 1, and the rounds t = 30. Take the Top-10 nodes from each algorithm as infective nodes and infect other nodes in networks, the final Top-10 nodes infection capacity of 12 algorithms in different colors are the average number of infected nodes F(t) obtained after 1000 iterations in 30 rounds.

As evident from the average number of infected nodes F(t) depicted in Fig. 7, the curves of 12 algorithms in 10 networks increases with the increase in rounds, and the growth rate is gradually slow. Among the 10 networks, in Euroroad, Usair, Friendships, and Protein networks, KLN has the best overall infection effect. In Karate, Dolphins, Powergrid, and CA-astroph networks, KLN is close to the optimal comparison algorithm. Meanwhile, in HepPh network, the overall performance of the 12 comparison algorithms is consistent, and the difference is not significant. In Football network, the overall effect of KLN is in the middle, while that of Enrenew is better. Because Enrenew restrains neighbor nodes after selecting influential nodes, it has better infection ability in the network that selects Top-*N* influential nodes (where *N* value is small), but it is not suitable for sorting nodes in the entire network. A comprehensive analysis shows that the Top-10 nodes in KLN can show better infection effects in most networks.

## Management strategies from case study

This paper conducts a case study on a real network, utilizing the 11 contrasting algorithms mentioned in the experiment and comparing them with the proposed KLN algorithm. The primary objective is to validate the applicability and accuracy of various algorithms in real networks. The selected experimental network is the Epi-2 epidemic propagation network^36^, situated within a 2-km radius in Bihar, India, spanning from January to June 2020.

**Figure 8:**
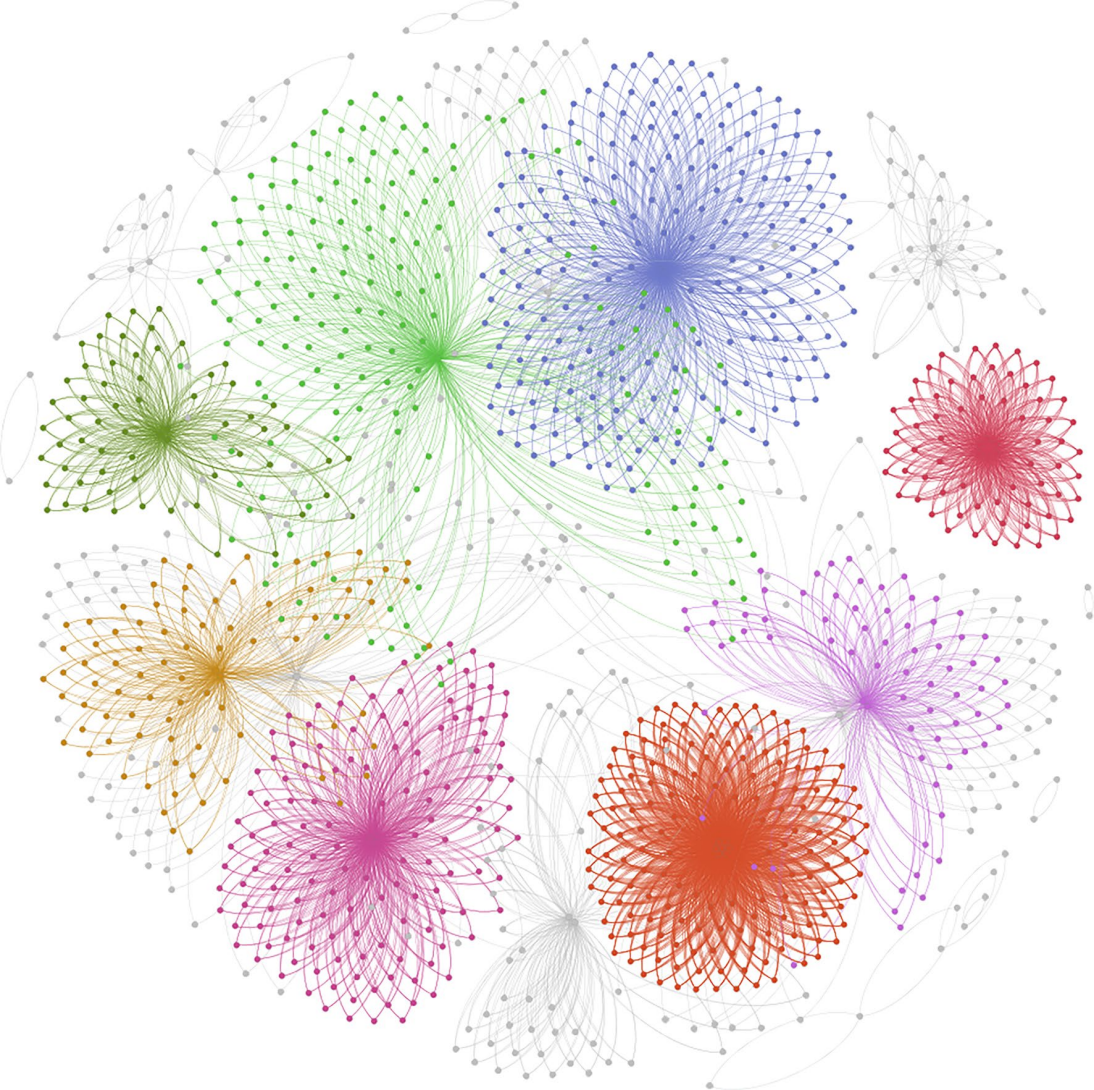
Epi-2 Network Model. In Epi-2 network, it is observed that the network encompasses 1204 nodes, forming 3193 contact chains and 6385 edges. The maximum degree in Epi-2 is 200, with an average degree of 5.303. The clustering coefficient is 0.056, and the modularity value is 0.831.

Epi-2 is formed by interactions among diverse individuals, constituting an undirected and unweighted complex network for epidemic propagation, as depicted in Fig. 8. The contagious nature of epidemic propagation contributes to distinct community characteristics within the node structure of this network, setting it apart significantly from the 10 networks studied in previous experiments.

### Top-30 nodes in epidemic propagation network

We ran the KLN algorithm and 11 compared algorithms in Epi-2 network, and compiled the Top-30 nodes, as illustrated in Table 7.

**Table 7:**
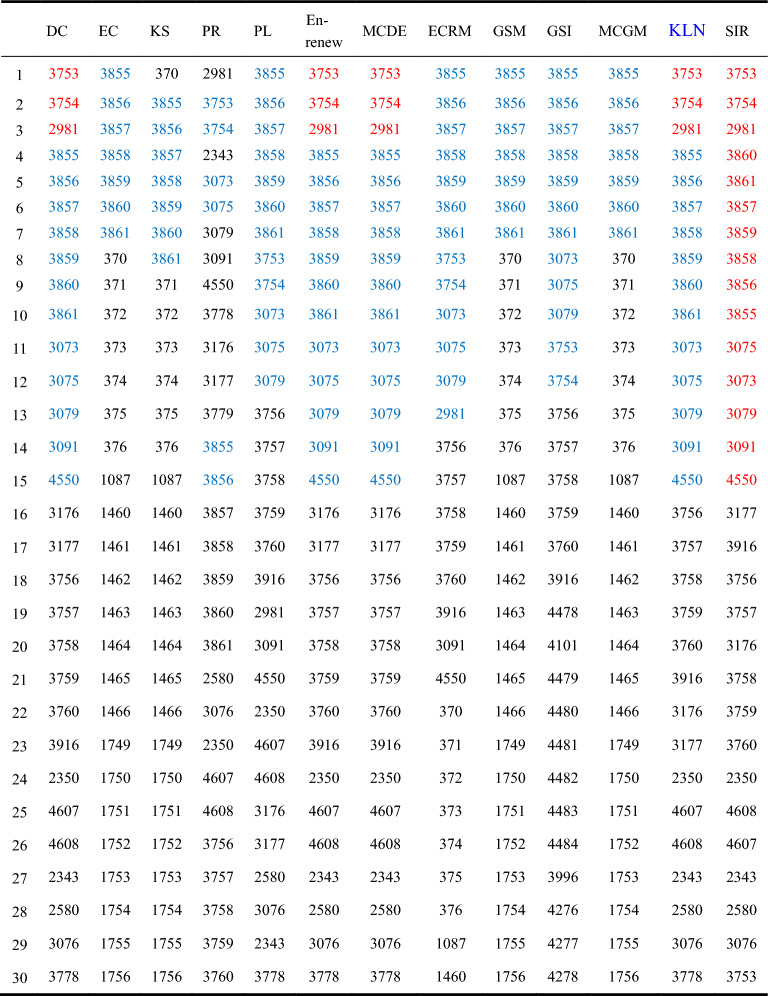
Top-30 nodes in Epi-2. Blue nodes in each compared algorithm represent the Top-15 nodes identified by SIR model, and the red nodes indicate that the order of these nodes is consistent with the order determined by SIR model.

In Table 7, we compared the Top-30 influential nodes selected by 12 algorithms with the results under SIR model. Since it is generally assumed that nodes ranked higher in the network play a more crucial role in propagation, we chose to analyze and compare in detail the first 15 nodes among the Top-30. Red nodes are used to indicate nodes whose order in key importance is identical to that of SIR model, while blue nodes are used to identify nodes ranking in the Top-15 under SIR model. From the Table 7, we can observe that in the KLN, DC, Enrenew, and MCDE algorithms, the first 15 nodes are entirely identical to those selected under SIR model. The order of the Top-3 nodes also matches precisely with the order under SIR model. However, beyond the Top-15 nodes, the rankings of these four algorithms start to differ from those of SIR model. Contrastingly, algorithms such as EC, Kshell, PL, GSI, GSM, and MCGM show relatively moderate performance, while the PR algorithm has the lowest Kendall value, indicating its unsuitability for application in this network.

### Kendall τ values of 12 algorithms in epidemic propagation network

**Figure 9:**
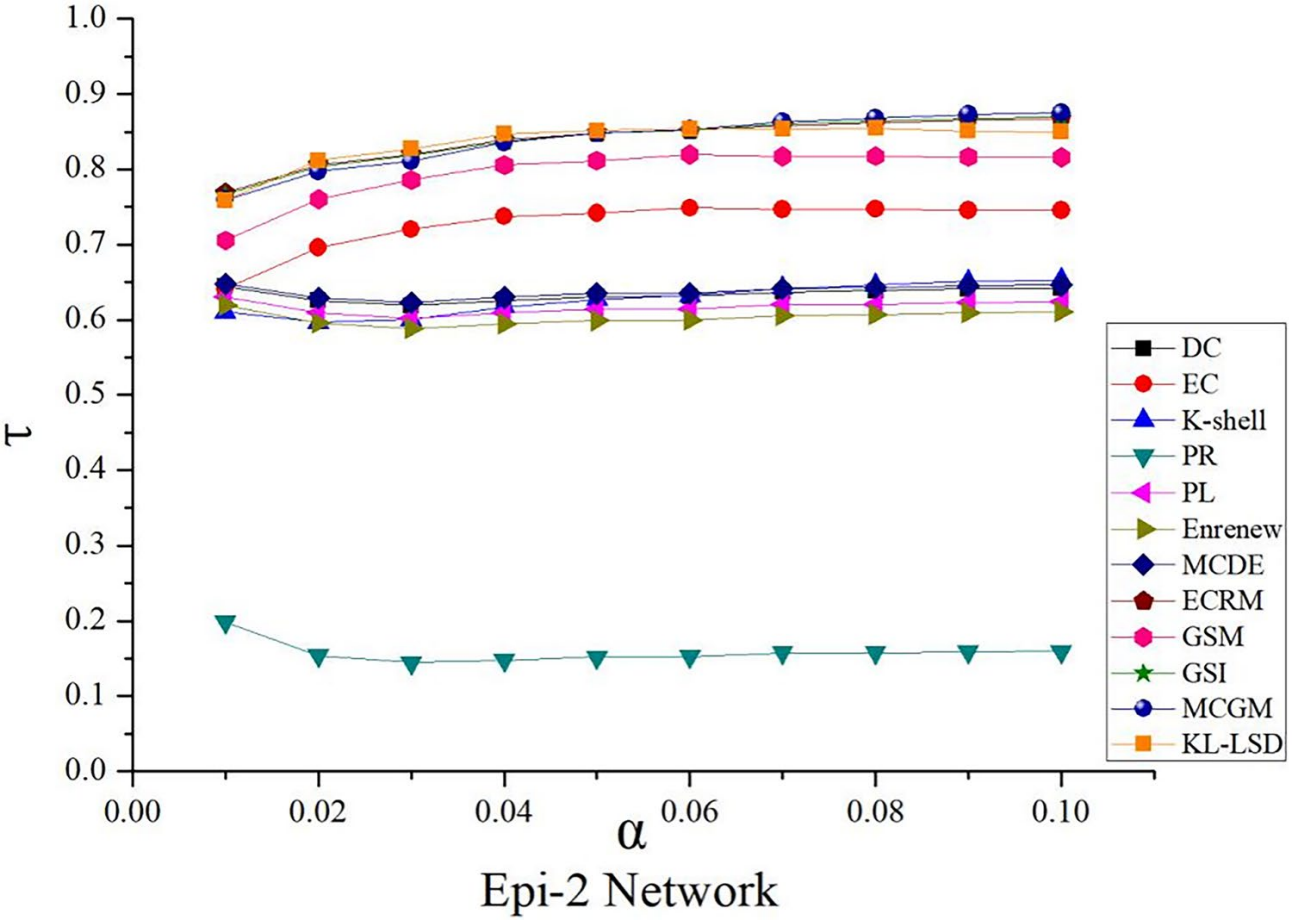
Kendall *τ* value in Epi-2 networks. The infection probabilities *α* is set as [0.01–0.1]. Kendall τ values of 12 algorithms in Epi-2 network in different colors are the average value obtained after 1000 iterations.

To further validate the accuracy of the algorithms, we conducted a comparative analysis of the Kendall τ values for the 12 algorithms, as shown in Fig. 9.

From Fig. 9, it can be observed that within the infection probability range of [0.01–0.1], algorithms with relatively high Kendall τ values include KLN, MCGC, GSI, and GSM. Among them, the KLN algorithm exhibits the highest Kendall τ values in the infection probability range of [0.01–0.06], surpassing the values of the other 11 algorithms, indicating its higher accuracy. On the other hand, the PL algorithm has the lowest Kendall τ value, while the remaining seven contrasting algorithms generally have values ranging from 0.6 to 0.7, showing a relatively stable distribution. Comparing this with the aforementioned table, although MCGC, GSI, and GSM algorithms demonstrate certain advantages in the overall ranking of influential nodes, their consistency with SIR model is relatively low among the Top-30 influential nodes. Therefore, it can be concluded that the KLN algorithm exhibits high accuracy in both the Top-30 nodes and the overall network nodes, demonstrating the effectiveness of KLN algorithm.

### The management strategies in epidemic propagation networks

**Figure 10:**
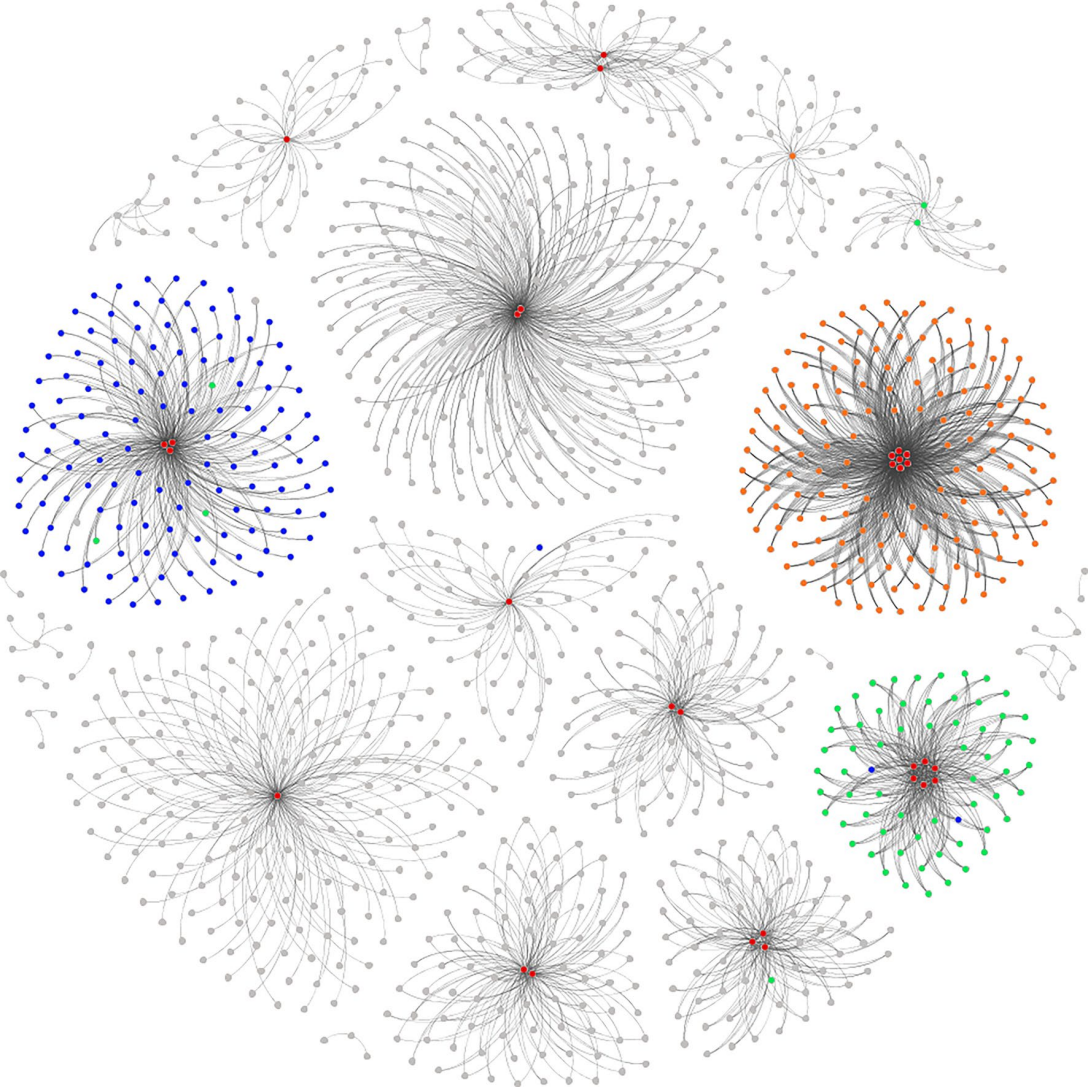
Influential nodes in the Epi-2 network. The red nodes are as the primary management nodes, the green nodes are as the secondary management nodes, the blue nodes are as the tertiary management nodes, and the yellow nodes are the quaternary management nodes, the remaining gray nodes are ordinary management nodes.

By conducting a deeper analysis of the modularity values of the Epi-2 network, the network characteristic illustrates certain community characteristics in a real epidemic propagation network. Connections between each community are relatively sparse, while nodes within each community are closely connected and share similar connection patterns. The nodes' importance values calculated by the KLN algorithm for the Top-30 nodes are highlighted in red on the graph. These 30 nodes are positioned at the center of each cluster, as shown in Fig. 10, indicating their significance. According to the sorting results, other nodes are labeled with different colors in Fig. 10: nodes ranked 31–183 are in green, nodes ranked 184–248 are in blue, nodes ranked 249–382 are in yellow, and the remaining nodes are in gray.

Therefore, through the analysis of the Epi-2 epidemic propagation network, it can be demonstrated that the KLN algorithm is effective in real-world networks. Furthermore, harnessing the identification results of KLN algorithm enables the development of effective management strategies for epidemic disease prevention and control within the network.

(1) In routine epidemic disease control, based on the importance values calculated by the KLN algorithm for each node, nodes of varying degrees of importance in the network can be categorized and managed. Vaccination strategies can be formulated with a focus on prioritizing nodes of high importance in the propagation network. For example, classifying nodes into different management categories, such as considering the top 30 nodes as a primary management focus, nodes ranked 31 to 183 as a secondary management group, nodes ranked 184 to 248 as a tertiary management group, and nodes ranked 249 to 382 as a quaternary management group. Nodes in higher categories should receive priority protection. Allocate more preventive resources to primary management objects, enhance the daily care awareness of this group, continuously monitor the health data of key individuals, and assess the effectiveness of vaccination to maximize the mitigation of epidemic disease spread, ensuring effective control of epidemic disease propagation upon the arrival of a virus.

(2) During an epidemic disease outbreak, rapid control of the top nodes in different communities within the network is crucial. Due to the community characteristics of propagation, each community can be managed separately, with a particular focus on regions with complex connection patterns. Simultaneously, leveraging the KLN algorithm to assess the influence of important nodes within each community on surrounding nodes. Based on the speed and probability of epidemic disease control and propagation, selecting a suitable *N* value to promptly isolate and control the top-N nodes in each community. This enables swift and precise control of critical nodes in the network, effectively curbing the spread of the epidemic disease.

## Conclusions

Finding influential nodes in complex networks is a crucial step in achieving successful operation of complex networks. It helps optimize system performance, improve resource utilization, enhance security, and achieve more efficient information flow control and processing. Most of the current algorithms that identify influential nodes rely on multi-attribute analysis of nodes within an unchanged network structure. However, these approaches fail to account for the damage influence of the removed node within the neighborhood, which is crucial for real-world networks. To solve this issue, this paper proposes KLN algorithm based on KL divergence model within the neighborhood to find influential nodes. The KLN begins by selecting a central node for removal, and computes the basic attributes of the network. Next, it utilizes KL divergence to measure the information entropy lost due to the alterations in neighborhood after the node removal. By combining network attributes, KLN provides an evaluation of the damage influence on the local network. Finally, the importance of nodes is determined by calculating the damage influence of all the one-top nodes in the removed node-centered local network structure. Compared with other 11 algorithms on 10 networks in terms of Top-10 nodes, correlation of Kendall τ, infection capacity of entire network, and infection capacity of the Top-10 nodes, KLN shows superiority over classical algorithms including DC, EC, and K-shell. It also outperforms new algorithms such as PR, PL, Enrenew, MCDE, ECRM, GSM, GSI, and MECG, showing better results and higher accuracy in most cases.

In future research, we will focus on studying complex network analysis based on federated learning. As networks formed by different data structures exhibit varied characteristics, we will delve into extracting and analyzing the features of each node in the complex system, leveraging the specific data traits of different nodes in federated learning combined with complex network theory. This endeavor aims to enhance the stability and reliability of network system operations.

## Data Availability

The datasets used and/or analyzed during the current study are available from the corresponding author on reasonable request.
